# Identification of shared neoantigens derived from frameshift mutations in the *APC* gene

**DOI:** 10.3389/fimmu.2025.1574955

**Published:** 2025-05-15

**Authors:** Peng Zhao, Clara Effenberger, Saki Matsumoto, Toshihiro Tanaka, Yusuke Nakamura, Kazuma Kiyotani

**Affiliations:** ^1^ Laboratory of Immunogenomics, Center for Intractable Diseases and ImmunoGenomics (CiDIG), National Institutes of Biomedical Innovation, Health and Nutrition (NIBN), Ibaraki-shi, Osaka, Japan; ^2^ Project for Immunogenomics, Cancer Precision Medicine Center, Japanese Foundation for Cancer Research, Tokyo, Japan; ^3^ Department of Human Genetics and Disease Diversity, Graduate School of Medical and Dental Sciences, Institute of Science Tokyo, Tokyo, Japan; ^4^ Bioresource Research Support Center, Institute of Science Tokyo, Tokyo, Japan

**Keywords:** cancer immunotherapy, frameshift indels, frameshift neoantigen, shared neoantigens, T cell receptor, TCR-engineered T cells, TCR-T cells

## Abstract

Recent advances of cancer immunotherapy have identified neoantigens as essential targets for personalized treatments. However, since neoantigens are generally unique in individual cancers, neoantigen therapies that are more broadly applicable are eagerly awaited. Shared neoantigens, derived from recurrent mutations found across multiple patients, are considered to be a challenging, but promising approach. Here we analyzed shared frameshift neoantigens derived from frameshift indels in TCGA exome sequencing data and identified 760 possible recurrent frameshift mutation clusters (FSCs) that share frameshifted open reding frames and premature stop codons. Among them, we investigated FSCs in the *APC* gene (APC-F2-1472* and APC-F3-1512*) and identified HLA-A*24:02-restricted frameshift neoantigen peptides that elicited specific CD8^+^ T cell responses. Subsequently we identified their corresponding T cell receptor (TCR) sequences and generated genetically-engineered T cells expressing these APC frameshift neoantigen-specific TCRs. These engineered T cells specifically recognized target cells presenting these neoantigens and cytotoxic activity against them, supporting the therapeutic potential of targeting APC frameshift neoantigens in cancer immunotherapy. This study provides compelling evidence for the development of neoantigen-based therapies targeting common frameshift peptides, offering a promising approach for more effective, relatively broadly applicable immunotherapeutic strategies that could benefit a subset population of cancer patients.

## Introduction

Recent advances of cancer immunotherapy have highlighted the critical role of T cells in cancer treatments by recognizing cancer-specific antigens. Neoantigens are one of the tumor-specific antigens, which are generated from somatic mutations and presented on HLA molecules on the cancer cells, and are recognized by T cell receptors (TCRs) expressed on the surface of T cells. Because of their high specificity to cancer cells, neoantigens are considered promising targets for immunotherapy. However, since neoantigens are generally patient-specific, developing more broadly applicable immunotherapeutic strategies has been expected. Shared neoantigens, derived from recurrent mutations found in multiple patients, offer a potential solution as they may facilitate broader clinical applications.

To date, several studies have reported shared neoantigens from mutations in the genes, such as *KRAS* and *TP53*, but these were mostly derived from the recurrent single-nucleotide variants (SNVs) (1–5). Through the comprehensive screening of shared neoantigens, we identified a shared neoantigen derived from FGFR3 Y373C mutation frequently observed in bladder cancer (6). However, accumulating information for more targetable shared neoantigens is essentially important to cover a broader patient population. Emerging evidence indicates that a high burden of frameshift insertions or deletions (indels) is significantly associated with favorable responses to immune checkpoint inhibitors, suggesting that frameshift indels can generate highly immunogenic neoantigens (7, 8). Frameshift indels create novel open reading frames (ORFs), resulting in unique peptide sequences downstream of the mutation site, which are really novel peptides that are not encoded by our normal genome. Even if the positions of indel sites are different, these mutations can sometimes yield partially-identical peptides in the novel ORF. We refer to these mutations as frameshift mutation clusters (FSCs), which can produce highly immunogenic shared neoantigens. However, only limited analyses have been conducted on shared neoantigens derived from frameshift indels (9, 10).

In this study, we aimed to identify frameshift indel-derived shared neoantigens commonly found among cancer patients using exome sequencing datasets in the Cancer Genome Atlas (TCGA) database. We identified two HLA-A*24:02-restricted shared frameshift neoantigens within these FSCs in the *APC* gene. Furthermore, we constructed APC frameshift neoantigen-specific TCR-engineered T (TCR-T) cells that showed specific recognition and cytotoxic activity against cells expressing the target frameshift neoantigens. Our finding highlights the therapeutic potential of shared neoantigen peptides generated from multiple frameshift mutations, offering a promising avenue for developing cancer immunotherapies that could be beneficial to a broader population of cancer patients.

## Materials and methods

### Prediction of potential shared frameshift neoantigens

**Figure 1:**
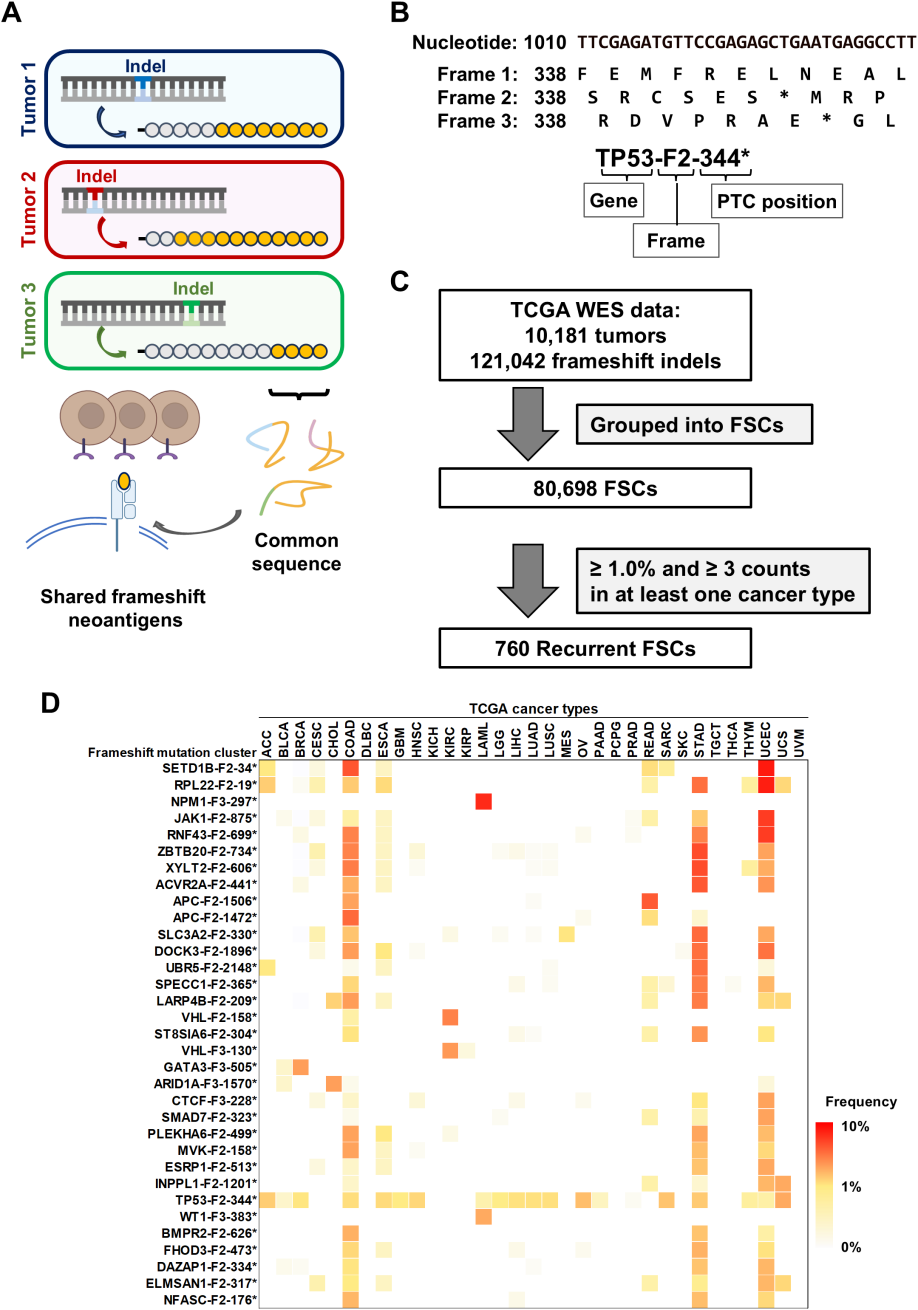
Identification of shared neoantigens derived from frameshift indels in cancer. **(A)** Overview of shared frameshift neoantigens. Frameshift indels create novel open reading frames (ORFs), resulting in unique peptide sequences (orange circles) downstream of the mutation site. Even if the positions of indel sites are different among cancers, these peptide sequences are partially-common and contain shared frameshift neoantigens. **(B)** The schema represents the labeling of frameshift mutation clusters (FSCs), which is based on gene name, shifted frame, and position of premature termination codon (PTC). Frame 1 is defined as reference ORF. Frames 2 and 3 are defined as the frames with 3n+2-bp insertion or 3n+1-bp deletion, and 3n+1-bp insertion or 3n+2-bp deletion, respectively. Asterisks (*) represent a PTC. **(C)** Workflow of the screening for recurrent FSCs by analyzing 121,042 frameshift indels within coding exons across 33 cancer types. By filtering a total of 80,698 FSCs by frequency, 760 recurrent FSCs were identified. **(D)** Heatmap of the frequency of FSCs in each cancer type in TCGA data. The FSCs present in more than 3% of cases in at least one cancer type are shown. Abbreviations of cancer types are based on the definition in TCGA (see also **Table 1**).

Somatic mutation data (MAF files) called using Mutect2 from TCGA, which consists of 10,181 samples across 33 cancer types, were downloaded from the NCI’s Genomic Data Commons (GDC) (11). For the 121,042 frameshift indels in the coding regions, downstream frameshifted nucleotide sequences were obtained based on Ensembl transcript data (release 79), and then translated into peptide sequences up to the point of a premature termination codon (PTC) (**Figure 1A**). Frameshift indels that shared ORF and terminated at same PTC position were grouped into FSCs. Frame 1 is defined as reference ORF. Frames 2 and 3 are defined as the frames with 3n+2-bp insertion or 3n+1-bp deletion, and 3n+1-bp insertion or 3n+2-bp deletion, respectively. FSCs were labeled based on gene name, shifted frame and position of PTC (**Figure 1B**). FSCs observed in more than 1.0% and in at least three different patients were further analyzed. Binding affinities of 8- to 11-mer peptides from these FSCs to HLA class I molecules were predicted using NetMHC 4.0 or NetMHCpan 4.1 (12, 13). Peptides for the shared neoantigen candidates were synthesized at a purity of >95% by Innopep Inc. (San Diego, CA, USA).

### Cell lines

C1R, Jiyoye, EB-3, 293 and retrovirus producer line Phoenix-AMPHO were purchased from the American Type Culture Collection (ATCC, Rockville, MD, USA). C1R, Jiyoye and EB-3 cells were cultured in RPMI1640 medium supplemented with 10% fetal bovine serum (FBS) at 37°C in a 5% CO_2_ incubator. 293 and Phoenix-AMPHO cells were cultured in EMEM and DMEM, respectively, both supplemented with 10% FBS at 37°C under 5% CO_2_ condition.

C1R cells stably expressing a single HLA class I, including C1R-A0201, C1R-0206, C1R-A1101, C1R-A2402, C1R-A3101 and C1R-A3303 established previously were used to investigate HLA-restricted T cell responses (6, 14). To evaluate the natural process from mutated protein to short peptide, we established 293 cells expressing mutated protein using a tandem minigene (TMG) as well as HLA-A24:02. 293 cells stably expressing a single HLA class I were obtained following the previously reported method (15). Briefly, endogenous HLA class I expression, all of the HLA-A, HLA-B and HLA-C, was knocked out using the CRISPR-Cas9 system to obtain 293HLA-KO cells. The 293HLA-KO cells were transfected with pCAGGS expression vector encoding HLA-A*24:02 cDNA and obtained stable transformant, 293-A24 cells. The 293-A24 cells were then transfected with pcDNA3.1 mammalian expression vector containing a TMG of four APC FSCs (encoding 33-, 51-, 41- and 28-mer peptides from APC-F2-1414*, APC-F2-1472*, APC-F2-1506* and APC-F3-1512* clusters, respectively) connected with cDNA of EGFP reporter to obtain 293-A24-APCTMG cells. Stable 293-A24-APCTMG cells exhibiting high expression both of HLA-A*24:02 and EGFP were sorted using cell sorter BD FACSMelody (BD Biosciences, San Jose, CA, USA).

### Induction of APC frameshift neoantigen-reactive CD8^+^ T cells from peripheral blood mononuclear cells

Induction of shared frameshift neoantigen-reactive CD8^+^ T cells was performed using PBMCs isolated from two HLA-A*24:02-positive healthy donors (Cellular Technology Ltd., Shaker Heights, OH, USA) following the protocols we previously reported (6, 16). Briefly, CD8^+^ T cells and CD8^-^ cells were separated using the Dynabeads CD8 Positive Isolation Kit (ThermoFisher Scientific, Carlsbad, CA, USA). Monocyte-derived dendritic cells (DCs) were generated from CD8^−^ cells using a plastic adherence method. By adding OK-432 at a final concentration of 0.1 KE/mL (Chugai Pharmaceutical Co., Tokyo, Japan), immature DCs were matured. The mature DCs were harvested on day 7 and pulsed overnight with 20 μg/mL of APC neoantigen long peptides (1472LP or 1512LP). For *de novo* T cell induction, autologous CD8^+^ T cells were co-cultured with the peptide-pulsed DCs for 7 days in AIM-V supplemented with 5% human AB serum (ABS), 10 ng/mL interleukin-7 (IL-7; R&D Systems, Minneapolis, MN, USA), and 24 IU/mL IL-2 (R&D Systems). After 7 days, induction of neoantigen-reactive T cells was assessed by IFN-γ Enzyme-Linked ImmunoSpot (ELISPOT) assay. The study protocol was approved by the Institutional Review Board of the Japanese Foundation for Cancer Research (2018-GA-1021) and National Institutes of Biomedical Innovation, Health and Nutrition (B2023-056). This study was conducted in accordance with the ethical principles outlined in the Declaration of Helsinki.

### ELISPOT assay

The ELISPOT assay was performed using the Human IFN-γ ELISpot PRO kit (Mabtech, Nacka Strand, Sweden). In brief, C1R cells expressing a single HLA type were pulsed with neoantigen peptides overnight and used as antigen-presenting cells. The neoantigen-reactive T cells induced above or TCR-engineered T cells were harvested and re-stimulated by co-culturing with the peptide-pulsed C1R cells for 24 h prior to the assay. As a positive control, 25 ng/mL phorbol-12-myristate-13-acetate (PMA) and 500 ng/mL ionomycin were added. Spots were captured and analyzed by an automated ELISPOT reader, ImmunoSPOT S6 (Cellular Technology Ltd.).

### TCR sequencing

To expand the ELISPOT-positive CD8^+^ T cells, cells were co-cultured with mitomycin C-treated feeder cells (Jiyoye and EB3 cells, each at 1×10^6^ cells/mL) in AIM-V containing 5% ABS, 100 IU/mL IL-2 and 10 ng/mL IL-21 (R&D Systems), followed by the activation with anti-CD3 and anti-CD28 antibodies (clones UCHT1 and CD28.2, respectively; BioLegend, San Diego, CA, USA) for 7 days at 37°C in a 5% CO_2_ incubator.

Peptide-loaded HLA-A*24:02 tetramers (Tetramer Shop, Kongens Lyngby, Denmark) and anti-human CD8α antibody (clone HIT8a; BioLegend) were used to stain neoantigen-specific T cell population, CD8^+^/tetramer^+^ T cells. Total RNA was extracted from the sorted CD8^+^/tetramer^+^ T cells, and the TCR sequencing library was constructed according to our previously established methods (17, 18). Briefly, cDNAs with universal SMART adapter were synthesized using the SMART cDNA library construction kit (Takara Bio, Shiga, Japan). TCRα and TCRβ cDNAs were separately amplified using reverse primers specific to the constant region of each chain and forward primer for the SMART adapter. The resulting PCR products were barcoded with the Nextera XT Index Kit (Illumina, San Diego, CA, USA) and sequenced on the Illumina MiSeq platform with 600 v3 reagents (Illumina) to obtain 300-bp paired-end reads. Obtained sequence reads were analyzed using Tcrip software (18).

### TCR-engineered T cells

The TCRα and TCRβ sequences linked by a furin-P2A self-cleaving peptide were synthesized after codon optimization for human codon usage to ensure efficient expression by GeneArt (ThermoFisher Scientific). These synthesized sequences were cloned into the pMXs retroviral vector (Cell Biolabs, San Diego, CA, USA). To enhance TCR expression on the T cell surface, mouse constant regions were introduced into both the TCRα and TCRβ chains, along with an additional disulfide bond to improve structural stability (19). To produce transient retroviral supernatants, Phoenix-AMPHO packaging cells were transfected with pMX-TCR expression vectors using TransIT-293 reagent (Mirus Bio, Madison, WI, USA). PBMCs from healthy donors were transduced as described previously (20). The expression of engineered TCRs was assessed using an anti-mouse TCRβ antibody (clone H57-597; BioLegend).

### Cytotoxicity assay

Cytotoxicity assays were performed by monitoring real-time cell quantity using impedance measurements, reported as the Cell Index (CI) using the xCelligence RTCA S16 Real-Time Cell Analyzer (Agilent Technologies, Santa Clara, CA, USA). Background impedance was first measured with cell culture medium alone. 293-A24-APCTMG cells were seeded at density of 25,000 cells/well, and once stable impedance readings indicated target cell attachment were observed (within approximately 20–25 h), TCR-T cells were added at an effector-to-target ratio of 10:1. Impedance was recorded at 15-min intervals, and the CI was normalized (nCI) to the values recorded just prior to T cell addition. The percentage of real-time cytotoxicity was calculated according to the following formula:


%Cytotoxicity=[Control nCI−(Experimental nCI−T cell nCI)/Control nCI]×100

Here, control nCI refers to the nCI of 293-A24-APCTMG cells without T cells as a baseline. Experimental nCI represents the nCI of 293-A24-APCTMG cells co-cultured with T cells, while T cell nCI represents the impedance contribution from T cells alone.

### Statistics

Statistical analyses were performed with Prism 8 (GraphPad software). Data are presented as means ± SD unless otherwise specified. A two-way ANOVA and Tukey’s HSD test were used for *post hoc* pairwise comparisons between groups at each peptide concentration in ELISPOT assays. Welch’s t-test was used to compare the data in cytotoxicity assays. A *P* value of <0.05 was considered as statistically significant.

## Results

### Screening of recurrent FSCs from TCGA exome sequencing data

It is reported that frameshift indels create novel ORFs, encoding unique peptide sequences which potentially contain neoantigens (7, 8). To screen for frameshift neoantigens that are shared among multiple cancer patients, we analyzed a total of 121,042 indels within coding regions from 10,181 exome sequencing data across 33 cancer types in TCGA database (**Figures 1A–C**). We classified these indels into a total of 80,698 FSCs based on common peptide sequences and further narrowed down them into 760 recurrent FSCs that were found in the frequency of more than 1.0% and in at least three patients (**Figure 1C**, **Supplementary Table 1**). Overall, the TP53-F2-344* cluster was the most prevalent in pan-cancer analysis, appearing in 20 different cancer types and representing approximately 1.0% (0.20%-3.5%) frequency of all the cases, followed by RPL22-F2-19* at 0.91%, RNF43-F2-699* at 0.87% and SETD1B-F2-34* at 0.82% (**Figure 1D**). In the analysis of frequent FSCs in each cancer type, we observed a high number of recurrent FSCs in several types of cancers, including colon adenocarcinomas, stomach adenocarcinomas and uterine endometrial carcinomas (**Table 1**). The SETD1B-F2-34* cluster was particularly prominent in uterine endometrial carcinomas, where it accounted for 9.1% of the patients, and it was also observed in 6.8% of the colon adenocarcinoma patients. The NPM1-F3-297* and WT1-F3-383* clusters were found in 8.4% and 3.5% of acute myeloid leukemia patients, respectively. Additionally, the ZBTB20-F2-734* and SETD1B-F2-34* clusters were present in 7.1% of stomach adenocarcinoma patients. In chromophobe cell renal carcinoma, the VHL-F2-158* and VHL-F3-130* clusters appeared in 5.1% and 4.2% of the patients, respectively.

**Table 1:** Summary of number of recurrent frameshift mutation clusters (FSCs) in 33 TCGA cancer types.

Cancer types^a^		Sample size	FSCs	Recurrent FSCs^b^
Adrenocortical carcinoma	(ACC)	92	433	30
Bladder urothelial carcinoma	(BLCA)	412	2,351	31
Breast invasive carcinoma	(BRCA)	986	4,211	107
Cervical squamous cell carcinoma and endocervical adenocarcinoma	(CESC)	289	2,779	103
Cholangiocarcinoma	(CHOL)	51	273	14
Colon adenocarcinoma	(COAD)	399	16,642	625
Lymphoid neoplasm diffuse large B-cell lymphoma	(DLBC)	37	132	0
Esophageal carcinoma	(ESCA)	184	1,588	119
Glioblastoma multiforme	(GBM)	393	2,527	16
Head and neck squamous cell carcinoma	(HNSC)	508	3,070	76
Kidney chromophobe	(KICH)	66	89	2
Kidney renal clear cell carcinoma	(KIRC)	336	2,638	31
Kidney renal papillary cell carcinoma	(KIRP)	281	1,858	14
Acute myeloid leukemia	(LAML)	143	656	4
Brain lower grade glioma	(LGG)	508	1,341	37
Liver hepatocellular carcinoma	(LIHC)	364	1,818	33
Lung adenocarcinoma	(LUAD)	567	5,549	44
Lung squamous cell carcinoma	(LUSC)	492	4,585	73
Mesothelioma	(MESO)	82	170	4
Ovarian serous cystadenocarcinoma	(OV)	436	4,880	30
Pancreatic adenocarcinoma	(PAAD)	178	406	17
Pheochromocytoma and paraganglioma	(PCPG)	179	109	0
Prostate adenocarcinoma	(PRAD)	495	1,293	44
Rectum adenocarcinoma	(READ)	137	1,380	98
Sarcoma	(SARC)	237	652	21
Skin cutaneous melanoma	(SKCM)	467	1,642	20
Stomach adenocarcinoma	(STAD)	437	15,959	615
Testicular germ cell tumors	(TGCT)	144	120	2
Thyroid carcinoma	(THCA)	492	1,355	3
Thymoma	(THYM)	123	265	23
Uterine corpus endometrial carcinoma	(UCEC)	530	21,188	639
Uterine carcinosarcoma	(UCS)	57	365	28
Uveal melanoma	(UVM)	80	64	0

a Abbreviations of cancer types are based on the definition in TCGA.

b Recurrent FSCs are defined as FSCs detected in more than 1.0% and in at least three patients.

**Figure 2:**
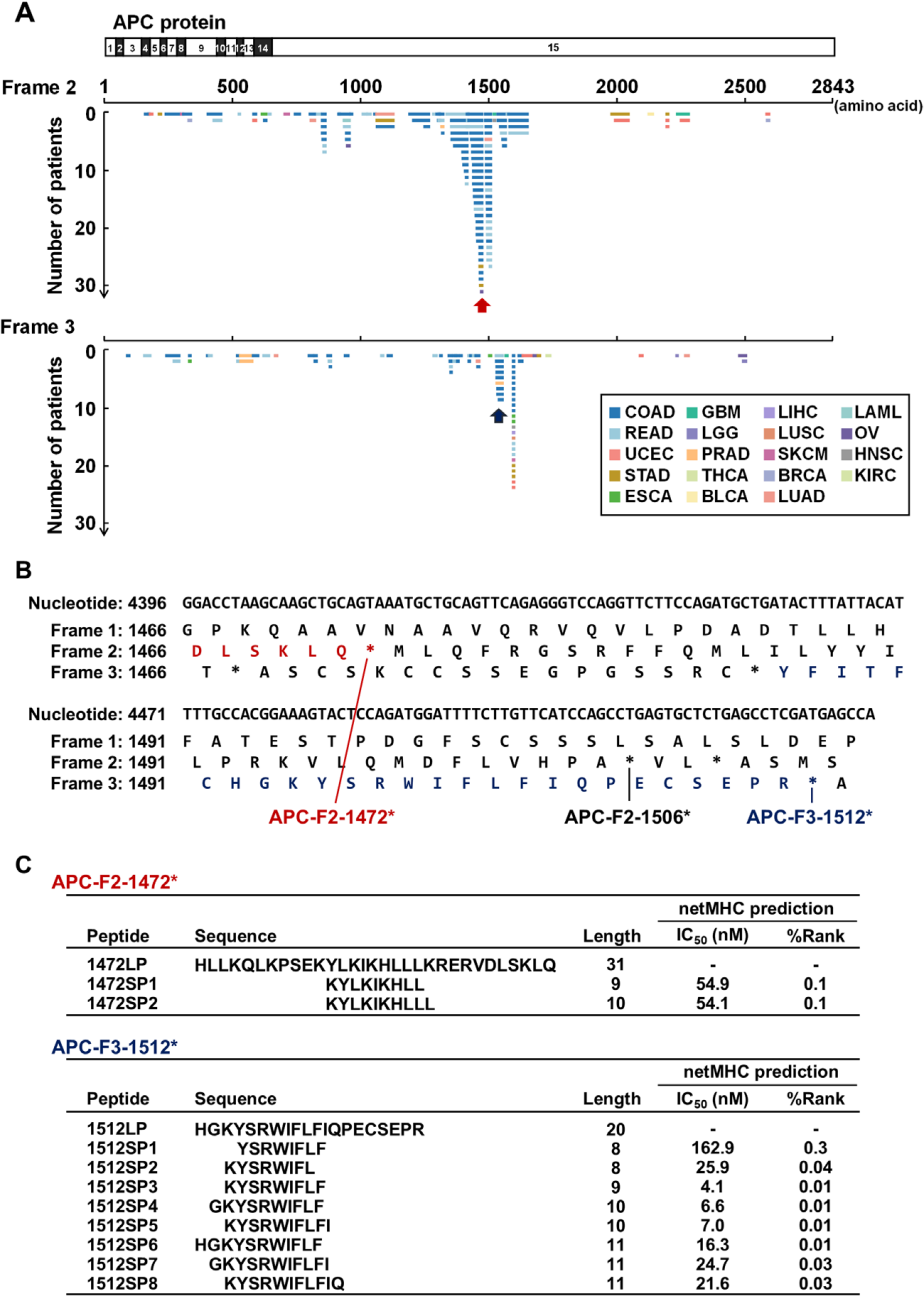
Shared neoantigen screening in frameshift mutation clusters in the *APC* gene. **(A)** Diagram of novel protein sequences generated by frameshift mutations in the *APC* gene. Frame 1 represents the reference protein sequence, while frames 2 and 3 represent alternative open reading frames (ORFs). Horizontal lines represent potential novel protein sequences generated by frameshift indels across patients. Red and blue arrows indicate APC-F2-1472* and APC-F3-1512* frameshift mutation clusters (FSCs), respectively. Abbreviations of cancer types are based on the definition in TCGA (see also **Table 1**). **(B)** Nucleotide and protein sequences around APC-F2-1472* and APC-F3-1512* clusters. Frame 1 is defined as reference ORF. Frames 2 and 3 are defined as the frames with 3n+2-bp insertion or 3n+1-bp deletion, and 3n+1-bp insertion or 3n+2-bp deletion, respectively. Asterisks (*) represent a premature termination codon. **(C)** Table summarizing the shared frameshift neoantigen short peptides (SPs) predicted within the APC-F2-1472* and APC-F3-1512* clusters. Long peptides (LPs) were designed to cover the SPs.

Consistent with previous findings highlighting the frequent occurrence of *APC* mutations (21, 22), we observed a high prevalence of FSCs within the *APC* gene in colon and rectal adenocarcinomas (**Figures 1D**, **2A**). We found eight APC FSCs (APC-F2-860*, APC-F2-1320*, APC-F2-1414*, APC-F2-1472*, APC-F2-1506*, APC-F3-1512*, APC-F3-1557* and APC-F2-1564*) in colorectal cancer (CRC) with a frequency of more than 1%, and conducted neoantigen predictions for nine HLA-A types, which are common in Japanese or Caucasians (**Supplementary Table 2**). Among the eight FSCs, our neoantigen prediction analysis found predicted neoantigens binding to several HLA types, such as HLA-A*02:01, HLA-A*03:01, HLA-A*11:01, HLA-A*24:02, HLA-A*31:01 and HLA-A*33:03 in five FSCs except for APC-F2-860*, APC-F2-1320* and APC-F3-1557*. We further analyzed the APC-F2-1472* (observed in 4.9%) and APC-F3-1512* (observed in 1.5%) clusters as potentially promising shared neoantigens, which could be presented by HLA-A*24:02 that is most common in the Japanese population (**Figures 2B, C**).

### Induction of CD8^+^ T cells reactive to APC shared frameshift neoantigens

**Figure 3:**
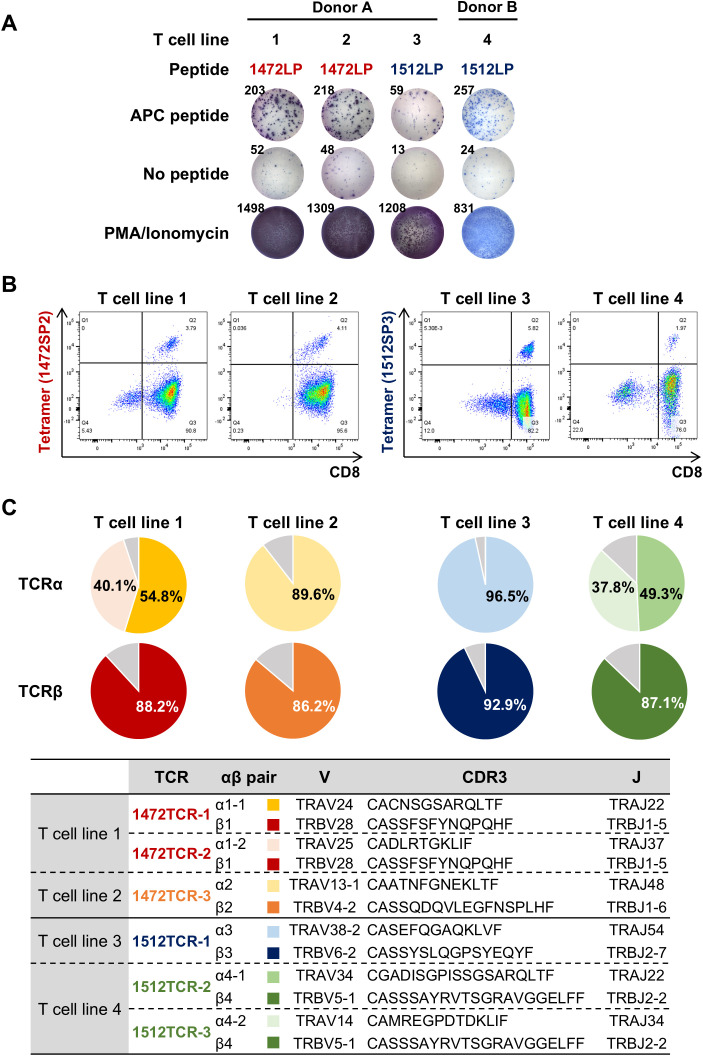
Identification of TCRs specific to APC frameshift neoantigens. **(A)** Induction of APC frameshift neoantigen-reactive CD8^+^ T cells from HLA-A*24:02-positive PBMCs. APC frameshift neoantigen-reactive T cells were induced by stimulating PBMCs with autologous dendritic cells pulsed with APC-F2-1472* and APC-F3-1512* long peptides (1472LP and 1512LP). IFN-γ ELISPOT assay was performed by re-stimulating induced neoantigen-reactive T cells by C1R-A24 cells pulsed with or without APC peptide, 1472LP or 1512LP (see also **Supplementary Figure 1**). PMA/ionomycin was used as a positive induction control. **(B)** Representative flow cytometry plots of peptide-HLA tetramers for expanded APC frameshift neoantigen-reactive CD8^+^ T cell lines. **(C)** Pie charts showing the frequencies of TCRα and TCRβ in sorted CD8^+^/tetramer^+^ T cells for APC frameshift neoantigen-reactive T cell lines 1 to 4.

To evaluate whether the predicted APC frameshift neoantigens can induce T cell responses, we designed long peptides which included the short neoantigen peptides predicted to be presented on HLA-A*24:02, and used them to perform T cell induction experiments using PBMCs obtained from two HLA-A*24:02-positive healthy donors. By stimulating PBMCs with autologous DCs pulsed with the 31-amino-acid long peptide 1472LP from the APC-F2-1472* cluster, we identified two distinct CD8^+^ T cell lines in donor A (T cell lines 1 and 2) that exhibited measurable IFN-γ secretion compared to the non-pulsed control, while no response was observed PBMCs in donor B (**Figure 3A**, **Supplementary Figure 1**). Using short peptides corresponding to the APC-F2-1472* cluster, we found that the T cell line 1 showed reactivity to a short peptide 1472SP2, but not to 1472SP1 although we were not able to examine the reactivity of the T cell line 2 due to the limited cell numbers (**Supplementary Figure 2**). When we stimulated PBMCs with the 20-amino-acid long peptide 1512LP from the APC-F3-1512*, one CD8^+^ T cell line (T cell line 3) from donor A showed reactivity to 1512LP, and similarly, another T cell population (T cell line 4) that responded to 1512LP with measurable IFN-γ secretion was obtained from donor B (**Figure 3A**, **Supplementary Figure 1**). We confirmed that T cell line 3 recognized a short peptide 1512SP3, which showed the strongest binding affinity to HLA-A*24:02 among the eight short peptides corresponding to a part of the APC-F3-1512* cluster (**Supplementary Figure 2**).

### Identification of APC shared frameshift neoantigen-specific TCRs

**Figure 4:**
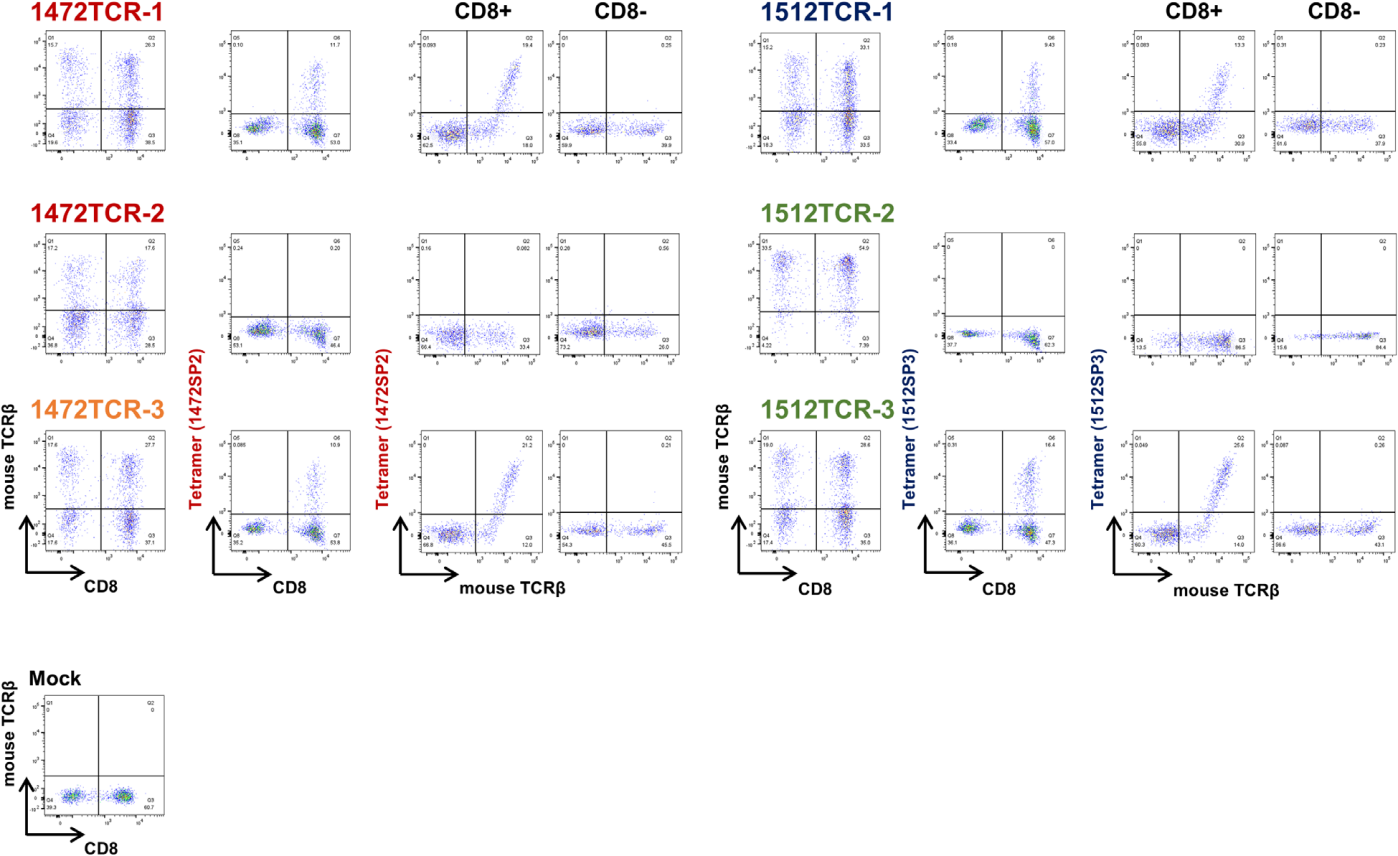
Determination of functional TCRαβ pairs for APC frameshift neoantigen-specific TCRs using TCR-T cells. The expressions of engineered 1472TCRs and 1512TCRs were confirmed using anti-mouse TCRβ antibody. The specific recognition of APC frameshift neoantigen was confirmed by peptide-loaded HLA-A*24:02 tetramers in CD8^-^ cells and CD8^+^ T cells.

To obtain TCR sequences of APC frameshift neoantigen-reactive CD8^+^ T cells, we expanded these four T cell lines by *in vitro* rapid expansion protocol, and then sorted by flow cytometry using 1472SP2- or 1512SP3-loaded HLA-A24:02 tetramers (**Figure 3B**), followed by next-generation sequencing of their TCRs (**Figure 3C**). Among the sorted CD8^+^ T cells from the four T cell lines, two T cell lines, 2 and 3, showed one predominant TCRα and TCRβ, which are likely to correspond to neoantigen-specific TCRα/TCRβ pairs. While T cell lines 1 and 4 exhibited two predominant TCRα clonotypes and one TCRβ clonotype; in T cell line 1, the two TCRα clonotypes (TCRα1–1 and TCRα1-2) were detected at frequencies of 54.8% and 40.1%, while in T cell line 4, TCRα4–1 and TCRα4–2 were present at 49.3% and 37.8% frequencies. It was previously reported that two different TCRα were sometimes expressed in a single T cell (23). Hence, we generated engineered T cells expressing two possible TCRα/TCRβ pairs for each of T cell lines 1 and 2, and examined which TCRα can recognize APC-F2-1472* and APC-F3-1512* frameshift neoantigens, respectively, using PBMCs isolated from healthy donors. 1472SP2-loaded HLA-A*24:02 tetramer detected 1472TCR-1 (TCRα1-1/TCRβ1)- transduced T cells, but did not recognize 1472TCR-2 (TCRα1-2/TCRβ1)- transduced T cells, suggesting that a heterodimer of TCRα1–1 and TCRβ1 is functional TCRαβ pair to recognize APC-F2-1472* frameshift neoantigen (**Figure 4**). Similarly, 1512SP3-loaded HLA-A*24:02 tetramer detected only 1512TCR-3 (TCRα4-2/TCRβ4), but did not detect 1512TCR-2 (TCRα4-1/TCRβ4), indicating the TCRα4–2 and TCRβ4 pair is a specific TCR for APC-F3-1512* frameshift neoantigen.

### Immunological activity of APC shared frameshift neoantigen-specific TCR-T cells

**Figure 5:**
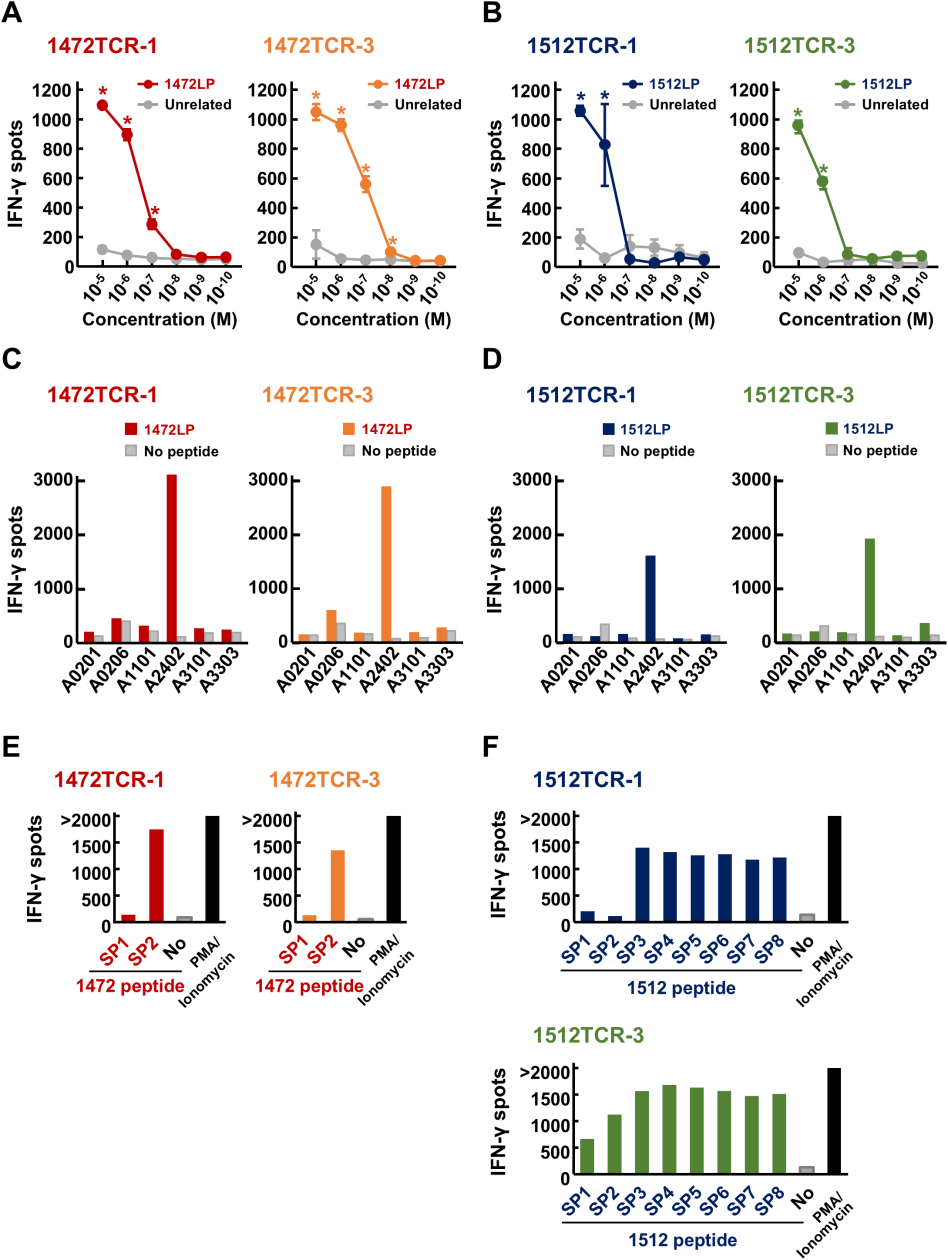
Immunological reactivity of APC frameshift neoantigen-specific TCR-engineered T cells. **(A, B)** Dose-dependent IFN-γ secretion from TCR-T cells after stimulated by C1R-A24 loaded with different concentrations of APC-F2-1472* and APC-F3-1512* long peptides (1472LP and 1512LP), or unrelated peptide. Data are presented as means ± SD (*n* = 3). Statistical significance was evaluated using Tukey’s HSD test. *P < 0.05. **(C, D)** HLA-restricted IFN-γ secretion from TCR-T cells after stimulated by C1R cells expressing various HLA types, pulsed with or without APC-F2-1472* and APC-F3-1512* long peptides (1472LP and 1512LP). PMA/ionomycin was used as a positive induction control. **(E)** Screening of functional short peptides within APC-F2-1472*. IFN-γ ELISPOT assay was performed by stimulating 1472TCR-1- or 1472TCR-3-engineered T cells by C1R-A24 cells pulsed with or without APC-F2-1472* short peptides (1472SP1 and 1472SP2). **(F)** Screening of functional short peptides within APC-F3-1512*. IFN-γ ELISPOT assay was performed by stimulating 1512TCR-1- or 1512TCR-3-engineered T cells by C1R-A24 cells pulsed with or without APC-F3-1512* short peptides (1512SP1 to 1512SP8).

To confirm the immune reactivity of the four functional TCRs (1472TCR-1, 1472TCR-3, 1512TCR-1 and 1512TCR-3), we further analyzed the TCR-engineered T cells. TCR-engineered T cells expressing 1472TCR-1 or 1472TCR-3 showed IFN-γ secretion specifically when co-culturing with C1R-A24 cells pulsed with corresponding 1472LP at a peptide concentration of 10^–7^ M, but not with unrelated 1512LP even at 10^–5^ M condition (**Figure 5A**). TCR-T cells expressing 1512TCR-1 or 1512TCR-3 reacted to 1512LP-pulsed C1R-A24 cells in a dose-dependent manner (**Figure 5B**). Moreover, we examined HLA-restriction of these TCRs using C1R cells overexpressing various HLA-A, including C1R-A0201, C1R-A0206, C1R-A1101, C1R-A2402, C1R-A3101 and C1R-A3303 (**Figures 5C, D**). IFN-γ production was predominantly detected when co-cultured with C1R-A2402 cells, confirming their HLA-A*24:02-restricted T cell responses.

To further confirm core short peptide sequences within the long peptides, which can be presented on HLA-A24:02 molecules, we performed IFN-γ ELISPOT assay using serial 1472 or 1512 short peptides (1472SPs or 1512SPs, respectively). Consistent to the results using induced CD8^+^ T cells, both TCR-T cells with 1472TCR-1 and 1472TCR-3 responded to 10-mer 1472SP2, but not to 9-mer 1472SP1, despite the latter lacking only a leucine at the C-terminus (**Figure 5E**). For the 1512SP peptides, TCR-T cells with 1512TCR-1 or 1512TCR-3 showed different recognition patterns; TCR-T cells expressing 1512TCR-3 reacted to all the eight short peptides (1512SP1 to 1512SP8), while TCR-T cells with 1512TCR-1 showed IFN-γ production for six short peptides (1512SP3 to 1512SP8) but showed no response to 1512SP1 and 1512SP2 (**Figure 5F**).

**Figure 6:**
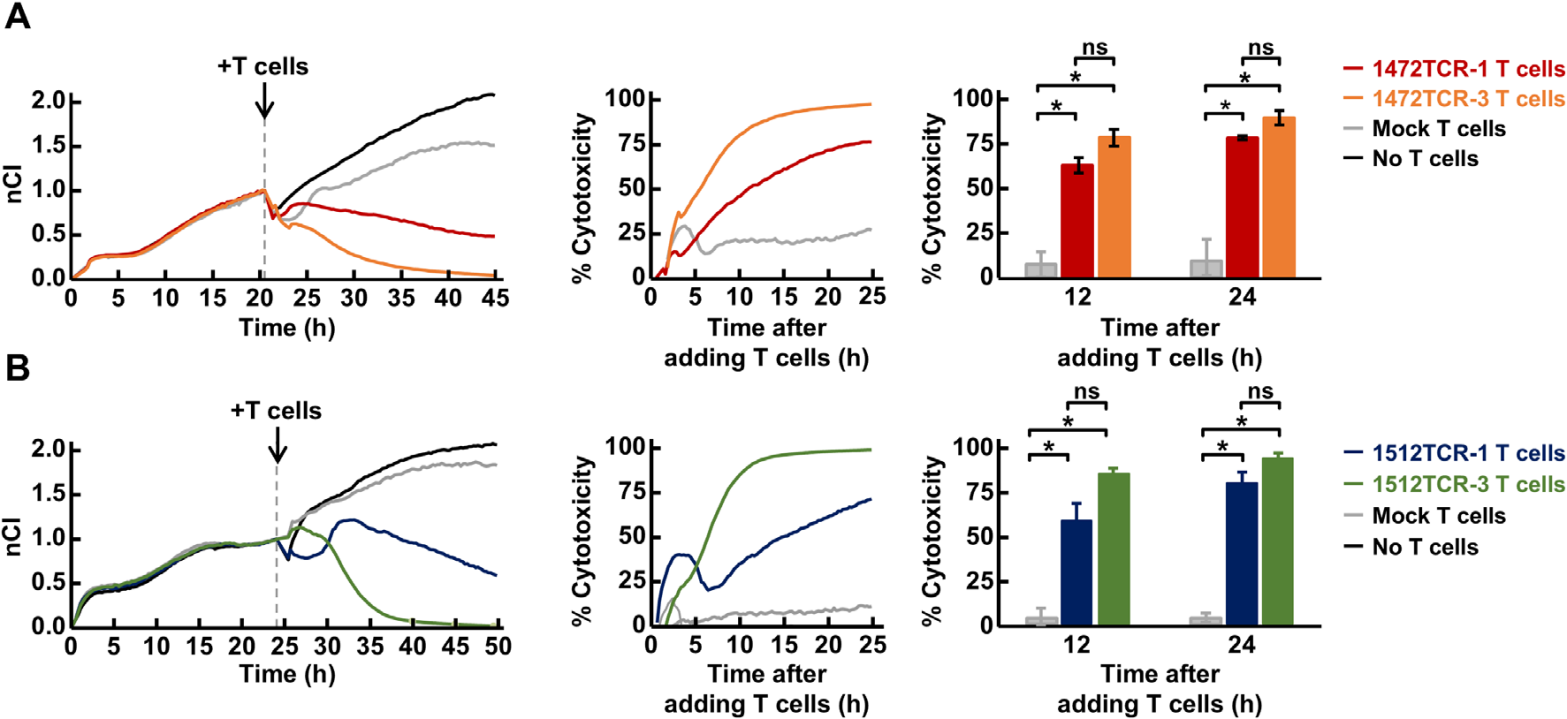
Cytotoxicity of APC frameshift neoantigen-specific TCR-engineered T cells. **(A, B)** Cytotoxicity assessed by real-time impedance measurement using the xCelligence RTCA S16 Real-Time Cell Analyzer. TCR-T cells expressing 1472TCRs **(A)**, 1512TCRs **(B)** or mock T cells which were not transduced with the TCRs, were co-cultured with 293-A24-APCTMG target cells at effector-to-target ratio of 10:1. Representative Normalized Cell Index (nCI) measured at 15-min intervals by impedance readings (left), the representative cytotoxicity calculated based on the difference in nCI between experimental and control conditions (middle), and cytotoxicity at 12 and 24 h time-points in three independent experiments (right). Data are presented as means ± SEM. Statistical significance was evaluated using Welch’s *t*-test. *P < 0.05.

To further evaluate the cytotoxic response of these TCR-T cells against target cells, we conducted an electrical resistance-based real-time cytotoxicity assay. Using 293-A24-APCTMG cells, expressing long peptides within the APC FSCs, including APC-F2-1472* and APC-F3-1512* clusters, as a target, we observed that all the four TCR-T cells demonstrated significant cytotoxic activities against the target cells although cytotoxic activities of these four TCR-T cells are different (**Figures 6A, B**). Notably, 1512TCR-3 and 1472TCR-3 showed the strongest cytotoxicity against target cells expressing corresponding neoantigens, eliminating all of the target cells within 15–20 h. In contrast, untransduced mock T cells exhibited minimal cytotoxicity to the 293-A24-APCTMG cells, demonstrating the specific cell lysis activity against the APC frameshift neoantigen-expressing cells. These results demonstrate that these two APC frameshift neoantigens were naturally processed in the cells and presented on HLA on the cell surface.

## Discussion

Cancer immunotherapy targeting shared neoantigens is a challenging, but promising approach because of their high cancer specificity and potential for broader therapeutic applicability. However, previous studies including ours have mostly focused on shared neoantigens derived from recurrent SNVs (1–6). In this study, through a comprehensive screening of shared frameshift indels, which can create novel ORF peptides common among multiple cancer patients, we identified 760 recurrent FSCs with the frequency of >1.0% and in at least three cancer patients (**Figure 1**, **Supplementary Table 1**). We observed a significant enrichment of recurrent FSCs in cancers with microsatellite instability-high (MSI-H) and mismatch repair-deficient (dMMR), particularly in colon adenocarcinomas, stomach adenocarcinomas and uterine endometrial carcinomas in TCGA data (**Table 1**). This observation aligns with previous reports documenting a higher prevalence of frameshift mutations in MSI-H/dMMR cancers due to the accumulation of indel errors during DNA replication (24). Among the top 10 genes we identified, seven were enriched in MSI-H tumors (25, 26). Notably, some of the frameshift indels in these genes, such as *SETD1B* and *RNF43* are known to encode immunogenic peptides capable of inducing specific CD8^+^ T cell responses in both healthy individuals and MSI-H patients (9, 25). This finding highlights the immunogenic potential of frameshift-derived neoantigens, which could be exploited for cancer immunotherapy.

In microsatellite stable (MSS) cancers, we identified NPM1-F3-297* cluster at frequency of 8.4% in acute myeloid leukemia, which consisted of only a single type of frameshift mutation, p.W288Cfs*12 (**Figure 1D**). This cluster has been confirmed as a target for immunotherapy in acute myeloid leukemia in previous studies (27–29). Shared neoantigens from the NPM1-F3-297* cluster were likely to be presented by HLA-A02 or HLA-A11, inducing specific immune responses and resulting in significant anti-tumor effects in mouse models. Additionally, we identified eight FSCs within the *APC* gene, which is frequently mutated (approximately 70-80%) in CRCs and nearly two-thirds of the mutations were frameshift mutations clustered in the mutation cluster region present in the last exon (21, 22). Among them, we found two FSCs, APC-F2-1472* and APC-F3-1512*, which generated immunogenic frameshift neoantigens presented on HLA-A24:02 (**Figures 2**-**6**, **Supplementary Table 2**). Since APC-F2-1472* and APC-F3-1512* were found in approximately 5.0% and 1.5% of CRC patients, respectively, frameshift neoantigens presented on HLA-A*24:02 from these clusters were calculated to be applicable to approximately 3.0% and 0.9% of Japanese CRC patients, based on the assumption of the HLA-A*24:02 allelic frequency of about 61% in the Japanese population (30). Furthermore, since HLA-A*24:02 is present in 5–20% frequency in the Caucasian population, this neoantigen-targeted therapy could potentially benefit a non-negligible portion of Caucasian patients as well. Considering the globally increasing trend of CRC incidence, with an estimated 2 million cases annually at present (31), targeting even a small fraction of patients through frameshift mutation-based immunotherapies could have a substantial impact.

While our *in vitro* experiments using C1R-A24 cells pulsed with long peptides and 293-A24 cells overexpressing TMG encoding the peptides demonstrate that APC frameshift neoantigen peptides are intracellularly processed and presented on HLA-A24:02 molecules on the cell surface, we were unable to fully recapitulate endogenous antigen presentation in CRC cells due to the lack of suitable CRC cell lines with the relevant *APC* mutations and HLA-A*24:02 expression. Furthermore, as any CRC patient samples with the relevant *APC* mutations and with an HLA-A*24:02 allele have not been found yet, we could not assess whether neoantigen-reactive T cells naturally exist or are activated in CRC patients. To address these limitations, future studies will need to focus on establishing appropriate CRC cell line models using patient-derived cancer cells and analyzing *in vivo* T cell responses in cancer patients. These efforts will help provide a more comprehensive understanding of the immunogenic potential of APC frameshift neoantigens in colorectal cancers.

In addition to APC-F2-1472* and APC-F3-1512*, we identified six additional FSCs in the *APC* gene such as APC-F2-1506*, APC-F3-1557* and APC-F2-1564*, which are present in the frequencies of 4.5%, 6.6% and 1.1% of CRCs, respectively, whose peptides were predicted to bind to other HLA types, such as HLA-A*02:01, HLA-A*03:01, HLA-A*11:01, HLA-A*31:01 and HLA-A*33:03 (**Supplementary Table 2**). Although these predictions need to be further validated experimentally, a broader range of APC-derived shared frameshift neoantigens would be applicable for immunotherapy if they work as shown in this study. Future studies could investigate the durability of TCR-T cell responses and the scalability of neoantigen-targeted therapies for broader application across different ethnic populations. Moreover, the methodology used to identify these clusters can be applied to other cancer types with frequent frameshift mutations.

In conclusion, this study highlights the potential of targeting shared frameshift neoantigens, particularly those derived from the *APC* gene, for cancer immunotherapy. The identification of frameshift mutation clusters such as APC-F2-1472* and APC-F3-1512* underscores their immunogenicity and applicability in specific patient populations, particularly those with CRC.

## Data Availability

The original contributions presented in the study are included in the article/**Supplementary Material**. Further inquiries can be directed to the corresponding author.
