# Supplementary material for: Identification of shared neoantigens derived from frameshift mutations in the APC gene
Source: Front Immunol. 2025 May 15;16:1574955. doi: 10.3389/fimmu.2025.1574955 (PMC12119627; doi:10.3389/fimmu.2025.1574955)
Supplement: Supplementary file 1 [file DataSheet1.pdf]

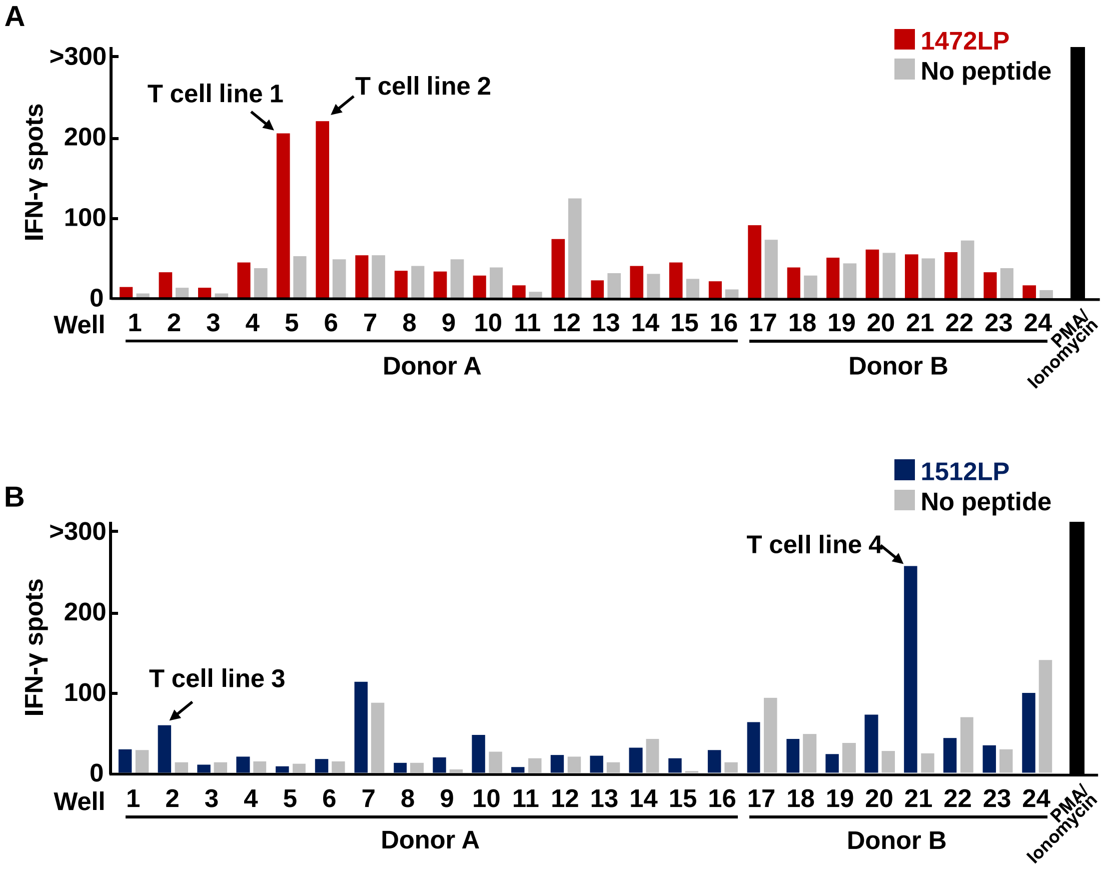

Supplementary Figure 1. Induction of APC frameshift neoantigen-reactive CD8$^+$ T cells from HLA-A*24:02-positive PBMCs.

(**A,B**) PBMCs were stimulated with autologous dendritic cells pulsed with long peptides, 1472LP (**A**) or 1512LP (**B**) to induce APC frameshift neoantigen-reactive CD8+ T cells. IFN-γ ELISPOT assay was performed by re-stimulating induced neoantigen-reactive T cells by C1R-A24 cells pulsed with or without 1472LP or 1512LP. PMA/ionomycin was used as a positive induction control. T cell lines 1 to 4 represented positive clones, related to **Figure 3**.
