# Supplementary material for: Identification of shared neoantigens derived from frameshift mutations in the APC gene
Source: Front Immunol. 2025 May 15;16:1574955. doi: 10.3389/fimmu.2025.1574955 (PMC12119627; doi:10.3389/fimmu.2025.1574955)
Supplement: Supplementary file 2 [file DataSheet2.pdf]

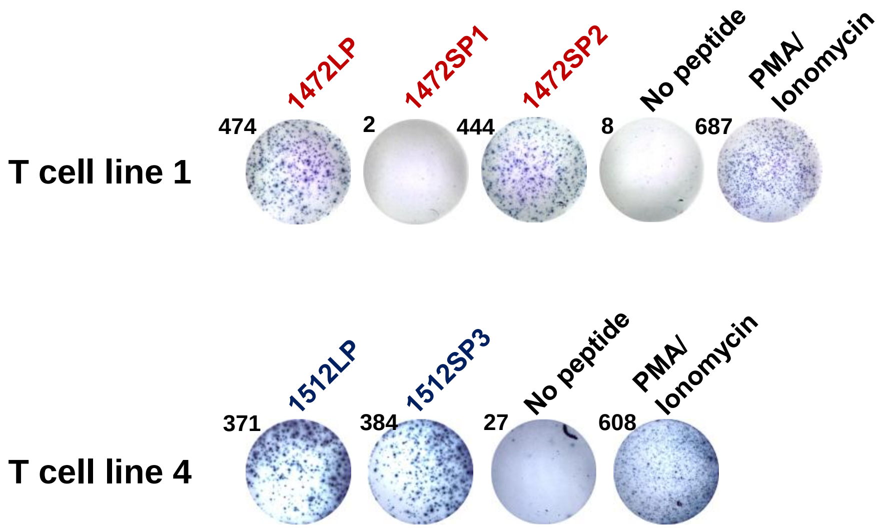

Supplementary Figure 2. Screening of immunogenic short peptides within APC long peptides.

IFN-γ ELISPOT assay for T cell line 1 from APC-F2-1472* and T cell line 4 from APC-F3-1512* stimulated by C1R-A2402 cells pulsed with or without 1472 peptides and 1512 peptides, respectively.
